# Maintenance of the Undifferentiated State in Myogenic Progenitor Cells by TGFβ Signaling is Smad Independent and Requires MEK Activation

**DOI:** 10.3390/ijms21031057

**Published:** 2020-02-05

**Authors:** Tetsuaki Miyake, Arif Aziz, John C. McDermott

**Affiliations:** 1 Department of Biology, York University, 4700 Keele Street, Toronto, ON M3J 1P3, Canada; tmiyake@yorku.ca (T.M.); Arif.Aziz@mnp.ca (A.A.); 2 Muscle Health Research Centre (MHRC), York University, Toronto, ON M3J 1P3, Canada; 3 Centre for Research in Biomolecular Interactions (CRBI), York University, Toronto, ON M3J 1P3, Canada; 4 Centre for Research in Mass Spectrometry (CRMS), York University, Toronto, ON M3J 1P3, Canada

**Keywords:** TGFβ, MEK, muscle, differentiation

## Abstract

Transforming growth factor β (TGFβ) is a pluripotent cytokine and regulates a myriad of biological processes. It has been established that TGFβ potently inhibits skeletal muscle differentiation; however, the molecular mechanism is not clearly defined. Previously, we reported that inhibition of the TGFβ canonical pathway by an inhibitory Smad, Smad7, does not reverse this effect on differentiation, suggesting that activation of receptor Smads (R-Smads) by TGFβ is not responsible for repression of myogenesis. In addition, pharmacological blockade of Smad3 activation by TGFβ did not reverse TGFβ’s inhibitory effect on myogenesis. In considering other pathways, we observed that TGFβ potently activates MEK/ERK, and a pharmacological inhibitor of MEK reversed TGFβ’s inhibitory effect on myogenesis, as indicated by a *myogenin* promoter-reporter gene, sarcomeric myosin heavy chain accumulation, and phenotypic myotube formation. Furthermore, we found that c-Jun, a known potent repressor of myogenesis, which is coincidently also a down-stream target of MEK/ERK signaling, was phosphorylated and accumulates in the nucleus in response to TGFβ activation. Taken together, these observations support a model in which TGFβ activates a MEK/ERK/c-Jun pathway to repress skeletal myogenesis, maintaining the pluripotent undifferentiated state in myogenic progenitors.

## 1. Introduction

TGFβ is the prototype of a large family of pluripotent cytokines with diverse effects on cellular proliferation, tumor growth, apoptosis, differentiation, fibrosis, anti-inflammation, and embryo development [1,2,3,4,5]. The competence of TGFβ cytokine signaling plays an important role in determining lineage acquisition in cells of mesenchymal origin, most notably by determining osteogenic or myogenic commitment [6]. The potency of TGFβ signaling at physiological levels in myogenic cells has been known for some time although dissection of the molecular pathway(s) mediating its cellular effects is still fragmentary.

The general canonical view that has been established for TGFβ signaling is that it binds to its cognate type II receptor, which facilitates receptor complex formation and activation of the cytoplasmic serine/threonine kinase activity of the type I receptor leading to phosphorylation of Smad2/3 (receptor regulated Smads: R-Smads). Phosphorylation of R-Smads at the C-terminal SXS motif results in association with the common Smad, Smad4, and translocation into the nucleus to regulate target gene transcription through complex interactions with heterogeneous transcription complexes [7,8]. While this pathway is pervasive in mediating TGFβ effects, a number of non-canonical aspects of TGFβ signaling have also been reported [9,10].

Previously, we observed that an inhibitory Smad (I-Smad), Smad7, potently counteracts Smad3 activation by TGFβ in a myogenic cell line. However, exogenous Smad7 was unexpectedly not able to prevent inhibition of muscle differentiation by TGFβ [11]. These observations indicate that TGFβ inhibits muscle differentiation through a Smad independent pathway. In further support for this idea, myostatin, a member of the TGFβ family and a regulator of skeletal muscle differentiation, also activates Smad2/3 by phosphorylation of the SXS motif of the R-Smads in a manner analogous to TGFβ. However, exogenous expression of Smad7 reverses the inhibitory effect of myostatin but not that of TGFβ [11]. Thus, several lines of evidence suggest that repression of myogenesis by TGFβ is mediated by a pathway distinct from the canonical R-Smad pathway. Here, we found that TGFβ activates MEK/ERK signaling. MEK activation subsequently represses the transcriptional activity of MyoD [12,13,14]. Importantly, a MEK-specific inhibitor, U0126 [15], reverses the inhibitory effect of TGFβ on myogenic differentiation, whereas pharmacological blockade of Smad3 signaling was without effect. These findings indicate that MEK, and not Smad3, activation is the primary mechanism underlying TGFβ’s inhibitory action on myogenesis. These observations reveal the involvement of a TGFβ-MEK pathway in maintaining myogenic precursor cells in the undifferentiated state and also place TGFβ at a strategic nexus to control the differentiation of pluripotent mesenchymal cells into different lineages.

## 2. Results

### 2.1. Inhibition of TGFβ Mediated Smad3 Phosphorylation Does not Reverse the Inhibitory Effect of TGFβ on Muscle Differentiation

**Figure 1:**
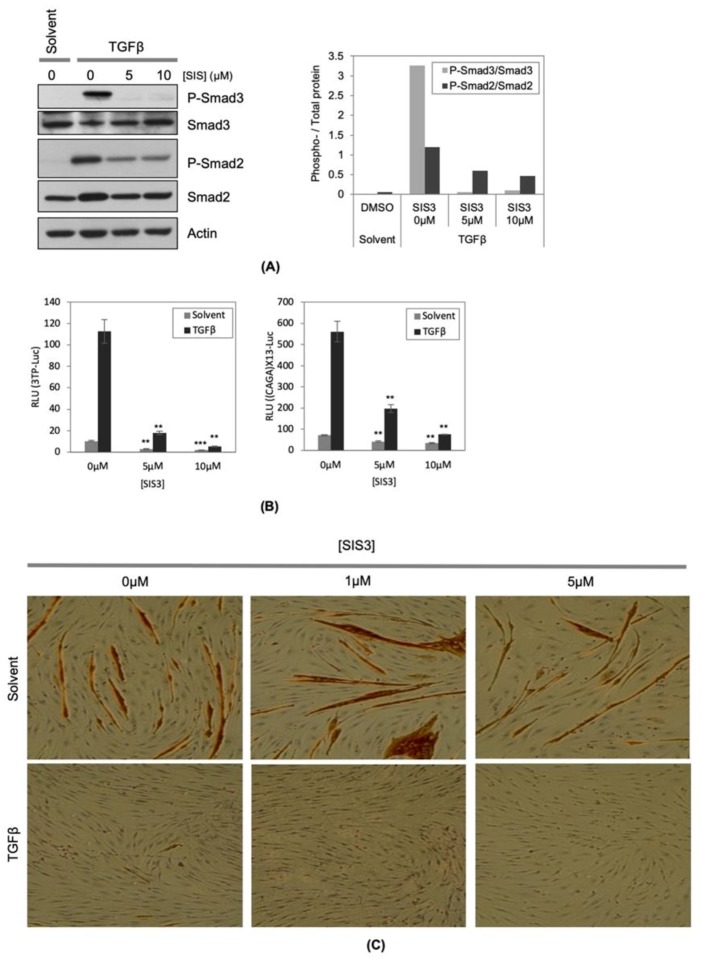
Inhibition of transforming growth factor β (TGFβ) mediated Smad3 phosphorylation does not reverse the inhibitory effect of TGFβ on muscle differentiation. (**A**) C2C12 cells were seeded onto cell culture plates at equal density and maintained in TGFβ (1ng/mL) with or without indicated concentrations of SIS3. Total protein samples were extracted from the cells and equal amounts of total protein (20 μg) were subjected to Western blotting analysis. The levels of indicated proteins were assessed by a standard immuno-blotting technique with a specific primary antibody. Actin indicates equal amounts of protein loading into each lane (left panel). Quantification of the band intensity of the P-Smad2 and P-Smad3, and total Smad2 and Smad3 in the left panel were measured by ImageJ, and the ratios of the band intensity of the phospho- to the corresponding total-protein band were graphed (right panel). (**B**) C2C12 cells were transfected with either 3TP-lux (left panel) or (CAGA)X13-luciferase reporter gene construct (right panel), and to monitor transfection efficiency, pCMV-β-gal construct was included in each condition. The transfected cells were maintained for 16 h in TGFβ (1ng/mL) with or without indicated concentrations of SIS3. Total protein samples were harvested with a luciferase lysis buffer. Luciferase activity (RLU: relative luciferase unit) in each condition was measured independently and normalized according to β-galactosidase activity. (*n* = 3, +/- SD). The *p*-value was calculated relative to the control by two tailed T-Test (** *p* < 1X10^−3^, *** *p* < 1X10^−5^). (**C**) C2C12 cells were seeded onto cell culture plates at equal density and maintained in TGFβ (1ng/mL) with or without indicated concentrations of SIS3 for 48 h. The cells were fixed and stained for muscle myosin heavy chain (MyHC) detection by immunochemistry. The photomicrographs are representative fields in each condition.

We previously found that although exogenous Smad7 strongly repressed Smad3 activation by TGFβ, the inhibition of muscle differentiation by TGFβ was not rescued by exogenous expression of Smad7 [11]. These observations suggested that TGFβ inhibits muscle differentiation in a R-Smad independent manner. To test this possibility, we inhibited Smad3 activation using a chemical inhibitor, SIS3 [16,17]. In agreement with previous observations, SIS3 strongly repressed TGFβinduced phosphorylation of Smad3 (Figure 1A). In the absence of SIS3, TGFβ potently enhanced the activity of 3TP-Lux and (CAGA)X13-Luc reporter genes, which are Smad3 dependent and TGFβ responsive reporter genes, respectively [18,19]. The activation of these two reporter genes by TGFβ was strongly repressed by SIS3 indicating the efficacy of SIS3 inhibition of Smad3 activation (Figure 1B). However, at the same concentration at which SIS3 strongly inhibits phosphorylation and activation of Smad3, SIS3 failed to reverse TGFβ’s inhibitory effect on muscle differentiation as assessed by myotube formation and myosin heavy chain (MyHC) accumulation (Figure 1C). In congruence with previous results, both SIS3 and Smad7 greatly reduced TGFβ induced Smad3 activity, but neither reversed TGFβ’s inhibitory effect on muscle differentiation (Figure 1C) [11]. Taken together, these observations strongly suggest that activation of Smad3 by TGFβ is insufficient for myogenic repression.

### 2.2. TGFβ Stimulates MEK Phosphorylation

We hypothesized that if Smad3 activation is insufficient to inhibit muscle differentiation, TGFβ must activate a non-canonical pathway to repress myogenesis. We did note that TGFβ-treated C2C12 cells reached a high density and survived better in differentiation medium (DM) (unpublished observation). This observation warrants further investigation into the possible effects of TGFβ on myoblast proliferation and survival. In other cell models, TGFβ has been observed to activate the MEK/ERK pathway [20,21], and we therefore assessed the MEK/ERK pathway as a potential target for TGFβ signaling in muscle cells. Previously, we documented that MEK activation is required for maintaining the undifferentiated state of myoblasts since a member of the IL-6 family, cardiotrophin-1 (CT-1), inhibits myogenesis through MEK activation [22]. Therefore, we treated C2C12 cells with recombinant TGFβ as well as CT-1 as a positive control to assess phosphorylation levels of MEK and Stat3.

**Figure 2:**
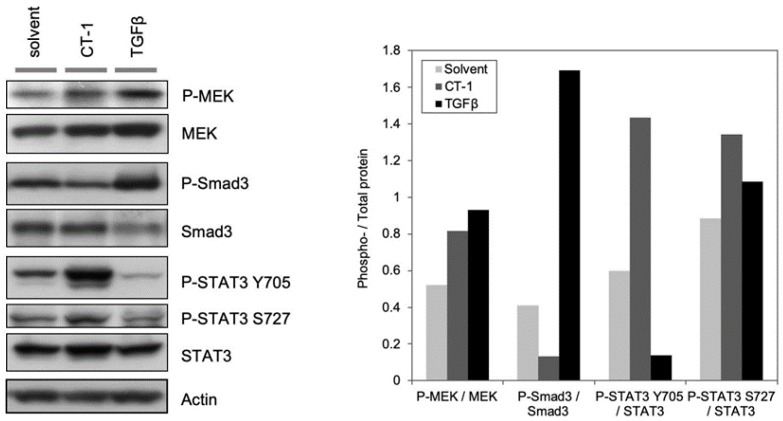
TGFβ stimulates MEK phosphorylation. C2C12 cells were seeded onto cell culture plates at equal density and maintained in TGFβ (1ng/mL), CT-1 (10ng/mL), or solvent. Total protein samples were extracted from the cells and equal amounts of total protein (20 μg) were subjected to Western blotting analysis. The levels of indicated proteins were assessed by a standard immuno-blotting technique with a specific primary antibody. Actin indicates equal amounts of protein loading into each lane (left panel). The experiment was done three times. Quantification of band intensity of the phospho- and corresponding total protein in the left panel were measured using ImageJ, and the ratios of the band intensity of the phospho- to the corresponding total protein band were graphed in each condition (right panel).

Assessment of the MEK signaling pathway activation by immuno-blotting with antibodies recognizing the total and phosphorylated forms of MEK revealed that phosphorylated MEK was enhanced in C2C12 cells treated with TGFβ (2ng/mL) compared to that in solvent treated control cells. TGFβ was more potent than CT-1 (10ng/mL) in terms of MEK activation based on the ratio of P-MEK to total MEK (Figure 2). Since we previously observed that CT-1 (10ng/mL) potently inhibits muscle differentiation in a MEK activation dependent manner [22], and the amount of phosphorylated MEK due to TGFβ treatment was higher than that of CT-1, we reasoned that TGFβ-mediated MEK activation in C2C12 cells may be sufficient to inhibit muscle differentiation. These results led us to postulate that TGFβ primarily inhibits myogenesis by activation of the MEK signaling pathway.

### 2.3. Inhibition of MEK Activation by a Pharmacological Inhibitor Partially Reverses the Inhibitory Effect of TGFβ on Muscle Differentiation

**Figure 3:**
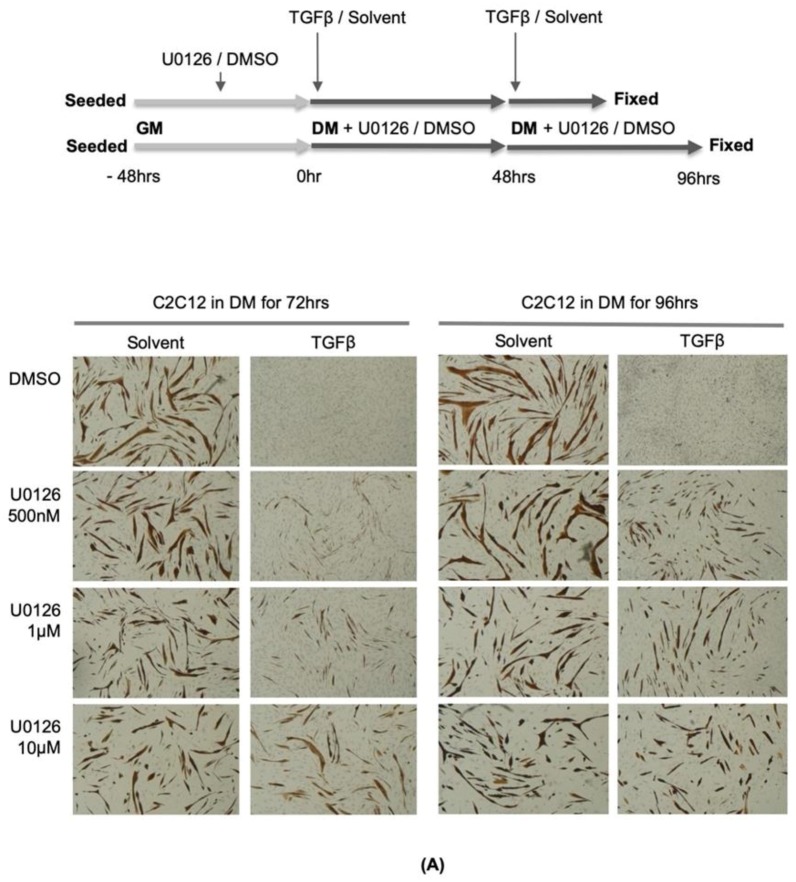

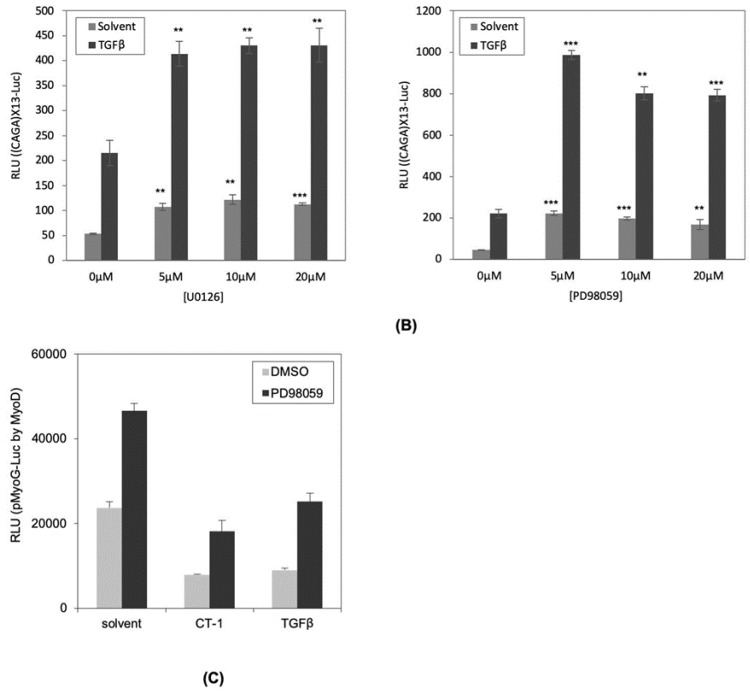
Pharmacological inhibition of MEK activation partially reverses the inhibitory effect of TGFβ on muscle differentiation. (**A**) C2C12 cells were seeded onto cell culture plates at equal density and maintained in TGFβ (1ng/mL) or solvent with or without indicated concentrations of U0126 for 72 (left panel) and 96 h (right panel). The cells were fixed and stained for muscle myosin heavy chain (MyHC) detection by immunochemistry (upper panel). The photomicrographs are representative fields in each condition (lower panel). (**B**) C2C12 cells were transfected with (CAGA)X13-luciferase reporter gene construct, and to monitor transfection efficiency, pCMV-β-gal construct was included in each condition. The transfected cells were maintained for 16 h in TGFβ (1ng/mL) or solvent with or without indicated concentrations of U0126 (left panel) or PD98059 (right panel). Total protein samples were harvested with a luciferase lysis buffer. Luciferase activity (RLU: relative luciferase unit) in each condition was measured independently and normalized according to β-galactosidase activity (*n* = 3, +/- SD). The p-value was calculated relative to the control by two tailed T-Test (** *p* < 1X10-3, *** *p* < 1X10-5). (**C**) C2C12 cells were transfected with myogenin promoter-luciferase reporter gene construct (pMyoG-Luc) with MyoD expression vector, and to monitor transfection efficiency, pCMV-β-gal construct was included in each condition. The transfected cells were maintained for 16 h in TGFβ (1ng/mL), CT-1 (10ng/mL), or solvent with or without indicated concentrations of PD98059. Total protein samples were harvested with a luciferase lysis buffer. Luciferase activity in each condition was measured independently and normalized according to β-galactosidase activity. (*n* = 3, +/- SD).

We next tested the possibility that prevention of MEK activation by a MEK-specific inhibitor might activate myotube formation and MyHC accumulation in the presence of TGFβ activation. As seen in Figure 3A, C2C12 cells cultured in DM for 72 h without exogenous TGFβ formed large multinucleated myotubes, and these myotubes accumulated a molecular differentiation marker (MyHC-brown stain). In the presence of exogenously added TGFβ in DM, as previously observed by us and several other groups [23,24,25], most of the cells maintained their mono-nucleated undifferentiated phenotype, and accumulation of MyHC was not observed (Figure 3B). This undifferentiated phenotype with exogenous TGFβ administration was essentially reversed by treatment of the cells with the MEK inhibitor, U0126, in a dose-dependent manner (Figure 3A). These data indicate that MEK is a key down-stream target of TGFβ signaling, and the activation of MEK contributes considerably to the inhibition of muscle differentiation.

Reversal of TGFβ mediated myogenic repression by MEK inhibition is not due to inhibition of Smad signaling since MEK/ERK signaling positively modulates R-Smad activity by phosphorylating the linker region of Smad2/3 [26,27,28,29]. In our experiments, MEK inhibition caused an increase in the activity of a TGFβ reporter gene consistent with the idea that MEK/ERK inhibition de-represses the R-Smads [27,29] (Figure 3B). Thus, MEK inhibition does not rescue myogenesis by repressing TGFβ induced R-Smad activation. These data further support the idea that R-Smad activation is absolutely unnecessary for myogenic repression by TGFβ.

### 2.4. MEK-Specific Inhibitors Reverse the Repression of the Myogenin Promoter by TGFβ

If MEK phosphorylation and subsequent nuclear accumulation are required for inhibition of muscle differentiation by TGFβ, we next tested whether MEK-specific pharmacological inhibitors might reverse TGFβ mediated repression of myogenic transcriptional activation properties [13] as quantified by the transcriptional activation of the *myog* gene, which is a critical myogenic target gene in the hierarchical control of myogenesis [30,31,32]. To assess this, a *myog* promoter-luciferase reporter gene (pMyoG-luc) was utilized. C2C12 cells were transfected with this reporter gene construct and a MyoD expression vector to fully activate the promoter. The transfected cells were treated with a MEK-specific inhibitor, PD98059, or DMSO (diluent), and TGFβ protein or its solvent. CT-1 treatment was included as a positive control in this analysis. TGFβ as well as CT-1 reduced MyoD driven *myog* promoter activation (Figure 3A). In the presence of PD98059 (10μM), neither TGFβ nor CT-1 was able to inhibit *myog* transcriptional activity efficiently suggesting the requirement for MEK signaling for myogenic repression by CT-1 and TGFβ (Figure 3C). Although we activated this promoter with exogenous MyoD expression, it is important to acknowledge that other myogenic regulators assembled on the *myog* promoter are also likely affected by MEK signaling (as discussed below). Therefore, these data indicate that TGFβ mediated MEK activation is required for the inhibition of a primary myogenic target gene, the *myog* gene.

### 2.5. TGFβ Signaling Modulates Myogenic Co-Activator and Co-Repressor Proteins

**Figure 4:**
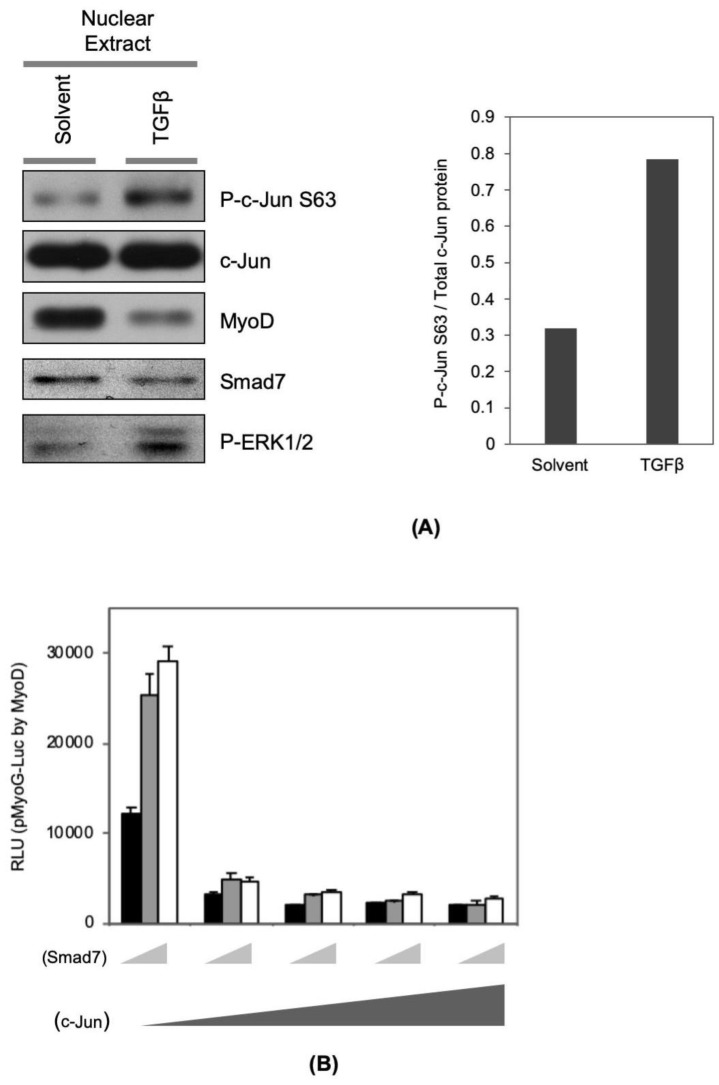
TGFβ signaling modulates MyoD co-activator and co-repressor proteins. (**A**) C2C12 cells were seeded onto cell culture plates at equal density and maintained in TGFβ (1 ng/mL) or solvent in DM for 16 h. Nuclear protein was extracted by using NE-PER^®^. The amount of indicated nuclear protein was visualized with standard Western blotting technique (left panel). The band intensity of P-c-Jun and total c-Jun were measured using ImageJ, and the ratio of the P-c-Jun to the c-Jun band intensity was graphed in the presence and absence of TGFβ (right panel). (**B**) C2C12 cells were transfected with myogenin promoter-luciferase reporter gene construct (pMyoG-Luc) with MyoD expression vector, and increasing amounts of c-Jun expression vector (0, 0.1, 0.4, 0.8, 1.6 μg) and combinations with Smad7 expression vector (0, 0.5, and 1 μg). In addition, to monitor transfection efficiency, pCMV-β-gal construct was included in each condition. The transfected cells were maintained for 16 h in DM. Total protein samples were harvested with a luciferase lysis buffer. Luciferase activity in each condition was measured independently and normalized according to β-Galactosidase activity (*n* = 3, +/- SD).

Previously, we reported that Smad7 can physically and functionally co-operate with MyoD in promoting myogenesis [11]. In this study, we found that Smad7 protein level is reduced by TGFβ treatment (Figure 4A). Since MyoD can bind to and activate the Smad7 promoter [11], this effect is likely mediated by interference with MyoD transcriptional properties and repression of this positive feed-forward loop by TGFβ. Secondly, we also found here that TGFβ signaling enhances the nuclear levels of phospho-MEK/-ERKs (Figure 4A, left panel) and phospho-c-Jun (Figure 4A, left and right panels), a well-established target of activated MEK signaling and a well characterized co-repressor of MyoD function [12,14]. Our data analyzing the *myog* promoter confirms this myogenic repression and also indicates that exogenous Smad7 expression cannot over-ride this repression (Figure 4A,B), which is consistent with Smad7′s inability to inhibit TGFβ mediated myogenic repression [11]. Therefore, the primary inhibition of the *myog* promoter results from downregulation of MyoD and myogenic co-activators such as Smad7, and induction of co-repressors such as c-Jun. We suggest that these molecular events constitute a mutually reinforcing network to lock the cells in an undifferentiated state.

## 3. Discussion

It is widely assumed that TGFβ inhibits skeletal muscle differentiation through activation of the canonical TGFβ/Smad3 pathway. However, we have made several key observations disputing this idea. First, Smad7, an inhibitory Smad, reversed the inhibitory effect of myostatin but not TGFβ on myogenesis even though Smad7 potently inhibits Smad3 activation induced by both myostatin and TGFβ [11]. Second, although a Smad3 specific inhibitor, SIS3, potently inhibited TGFβ-induced Smad3 phosphorylation and subsequent activation of TGFβ/Smad3 dependent gene expression, it did not reverse the inhibitory effect of TGFβ on myogenesis. Third, a MEK inhibitor reverses TGFβ’s inhibitory effect on myogenesis, which suggests a non-canonical pathway for myogenic repression. Fourth, nuclear Smad7, which is incapable of inhibiting the TGFβ/Smad3 “canonical” pathway, is sufficient to enhance myogenesis [33]. These observations clearly indicate that TGFβ inhibits muscle differentiation independent of activation of R-Smads.

### 3.1. Is MEK Activation a Common Effector of TGFβ Family Cytokines?

TGFβ is known to activate the MEK/ERK pathway [20,34], and we recently found that this MAPK pathway plays an important role in maintenance of the undifferentiated state of myogenic precursor cells by CT-1 [22], and now in the current study, by TGFβ. Interestingly, it has been documented that BMP4, which is a known inhibitor of myogenic cell specification during embryo development [35] and a member of the TGFβ superfamily, induces neuronal differentiation in a Ras/ERK dependent manner [36]. In the current study, we found that MyoD levels are decreased due to TGFβ treatment, which likely contributes to the repression of myogenesis. In addition, the increase observed in the active form of c-Jun is also relevant since this has previously been shown to post-translationally inhibit MyoD function [12,14]. The reduction in Smad7 levels are also relevant in view of reports implicating Smad7 in regulation of C2C12 terminal differentiation [11,33]. Moreover, we have also recently documented that Smad7/MyoD are components of a large myogenic protein complex assembled on the regulatory region of some myogenic genes [37]. Therefore, multiple changes in these myogenic regulators are mediated by TGFβ-MEK/ERK activation.

Thus, these observations suggest the possibility that TGFβ cytokines commonly invoke MEK activation to restrict or promote lineage acquisition depending on the cellular context. Further analysis of this idea is therefore warranted in the search for molecular regulators of cellular programming and re-programming.

A detailed analysis of Smad-independent TGFβ target genes may be enlightening in documenting these non-canonical effects of TGFβ signaling. Consistent with our observations, it was reported that *c-jun*, *junb*, and *smad7* genes are regulated by TGFβ in a Smad4-independent manner [38]. These observations suggest that regulation of *c-jun*, *junb*, and *smad7* gene expression by TGFβ would not be affected by inhibition of R-Smad activity, although this idea remains to be tested. Furthermore, exogenous Smad7 expression could not reverse TGFβ’s inhibitory effect on insulin-like growth factor binging protein-5 (IGFBP5) synthesis in C2C12 myoblasts (MBs) [39].

### 3.2. TGFβ Signaling in Muscle Pathologies

It has been reported that TGFβ signaling is increased in a number of muscle pathologies [40,41]. For example, canonical TGFβ signaling acutely increases when dystrophic muscle is stimulated to contract leading to fibrosis and muscle dysfunction in both mice and flies [40]. TGFβ1 levels increase rapidly in skeletal muscle after injury [42]. The post-natal muscle dysfunction observed in a number of studies has been largely attributed to the excessive fibrosis that occurs due to hyperactivated TGFβ signaling [43]. No doubt this is a major contributor to the pathology, however, it is tempting to speculate that an additional consideration, based on our data, is that the muscle progenitor cells (satellite cells) in diseased or injured muscle are inhibited from differentiating by TGFβ signaling, which might also contribute to muscle atrophy, inefficient repair and dysfunction. One study also indicated that a TGFβ-dependent conversion of muscle progenitors to a pro-fibrotic myofibroblast phenotype may also occur [43]. Chronic failure to differentiate progenitor cells in disease contexts could result in a progressive loss of contractile muscle mass—a fundamental hallmark of cachexia, which has also been linked with excessive TGFβ signaling [41].

The identification of MEK as a potent effecter of TGFβ signaling in myogenic cells will allow this pathway to be manipulated pharmacologically in a variety of contexts. Inhibiting this pathway with neutralizing antibodies or small molecule inhibitors could prove efficacious in muscle pathologies. Also, programming of multipotent mesenchymal cells or induced pluripotent stem cells may benefit from the characterization of signaling pathways that can efficiently repress or promote specific differentiation pathways in order to allow systematic programming and/or manipulation of cell identity and differentiation status.

In summary, we have characterized the mechanism of TGFβ’s inhibitory effect on myogenic cell differentiation at the molecular level. TGFβ-mediated repression of myogenesis is primarily dependent on MEK activation. Unexpectedly, TGFβ effects on myogenesis are unequivocally independent of R-Smad activation. Molecular dissection of TGFβ effects on myogenesis will allow further insights into its role during development and post-natal physiology and pathology of skeletal muscle.

## 4. Materials and Methods

### 4.1. Plasmids

Smad7 and Smad7T expression vectors were described previously [11]. An activated (ΔN3 S218D/S222E) human MEK1 expression construct was a gift from A. Natalie [44]. p3TP-Lux reporter construct was from J. Wrana (University of Toronto; Program in Molecular Biology and Cancer, Samuel Lunenfeld Research Institute, Mount Sinai Hospital; Toronto, Canada). (CAGA)X13-Luc reporter construct was generated by insertion of 13X (CAGA) sequence from the *pai-I* promoter [19] followed by a *c-fos* minimal promoter in the pGL3-basic (Promega; USA) luciferase reporter vector. pCMV-β-galactosidase have been described elsewhere [11]. The *myogenin* promoter region was excised from pMyoG-luc by *SacI*/*Bgl II* digestion. The resultant 1152bp fragment was inserted at the SacI/*Bgl II* sites of pGL4-10 vector (Promega, USA). The dsRed2-N1 expression vector was purchased from Clontech Laboratories; USA. All constructs used in this study were verified by DNA-sequencing (York University Molecular Core Facility; Toronto, Canada).

### 4.2. Antibodies

The primary antibodies used in this study were obtained from Santa Cruz Biotechnology; USA; MyoD (C-20), Actin (I-19) from Cell Signaling Technology; MEK1/2 (9122), phospho-MEK1/2 (Ser217/221) (9121), STAT3 (9132), phospho-STAT3 Y705 (9135), S727 (9136), Smad3 (9513), phospho-Smad3 (9514), Smad2 (3122), and phospho-Smad2 (3101) from DakoCytomation; USA; MyoD1 (clone:5.8A; M3512).

### 4.3. Cell Culture

C2C12 myoblast were obtained from the American Type Culture Collection; USA and cultured in growth medium (GM) consisting of 10% fetal bovine serum (FBS) (HyClone; Canada) in high-glucose Dulbecco’s modified Eagle’s medium (DMEM) (Gibco, USA) supplemented with 1% penicillin-streptomycin (Gibco; USA) at 37°C and 5% CO_2_. Myotube formation was induced by replacing GM with differentiation medium (DM), which consisted of 2% horse serum (Atlanta Biologicals; USA) in DMEM supplemented with 1% penicillin-streptomycin. For TGFβ or CT-1 treatment, recombinant human TGFβ (R&D system; USA; 240-B) or CT-1 (R&D system; 438-CT) was resuspended with solvent (4mM HCl, 0.1% bovine serum albumin (BSA)) and added into the media. For myotube formation assays, DM with TGFβ (1ng/mL) or CT-1 (10ng/mL) was replenished every 2 days. Inhibitors (PD98059 (9900), U0126 (Cell Signaling Technology, Canada; 9903), and SIS3 ((2E)-1-(6,7-Dimethoxy-3,4-dihydro-1H-isoquinolin-2-yl)-3-(1-methyl-2-phenyl-1H-pyrrolo[2–b]pyridin-3-yl)-propenone hydrochloride (Sigma-Aldric;, Canada, S0447) were resuspended with DMSO and added into the cell culture media for 30 min prior to adding TGFβ.

### 4.4. Microscopy

Phase contrast photomicrographs were obtained using an epifluorescence microscope (Axiovert 35; Carl Zeiss MicroImaging; Canada), with appropriate phase and filter settings, and either 4X NA 0.10 or 10X NA 0.25 Achrostigmat objective lenses. Images were recorded with a digital camera (Canon, EOS D6; Japan).

### 4.5. Nuclear Protein Extraction

Nuclear proteins were extracted from the cells by NE-PER^®^ kit (Pierce) according to the manufacturer’s protocol.

### 4.6. Western Blotting Analysis

Total cellular protein extracts were prepared in NP-40 lysis buffer (0.1 % NP-40, 150 mM NaCl, 1 mM EDTA, 50 mM Tris-HCl pH 8.0, 1 mM sodium vanadate, 1 mM PMSF, supplemented with a protease inhibitor cocktail (Sigma; Canada, P-8340)). Protein concentrations were determined by a standard Bradford assay (BioRad; USA). Equivalent amounts of protein were resolved by SDS-PAGE gels, followed by electrophoretic transfer to an Immobilon-P membrane (Millipore; Canada) as directed by the manufacturer (Millipore). Blots were incubated with the indicated primary antibody in 5% milk in PBS or Tris buffered saline (TBS)-T (10mM Tris-HCl pH8.0, 150mM NaCl, 0.1% Tween-20) or 5% Bovine serum albumin (BSA) in TBS-T according to the manufacturer’s protocol at 4°C overnight with gentle agitation. After washing briefly, the blots were incubated with the appropriate HRP-conjugated secondary antibodies in 5% milk in PBS or TBS-T at room temperature according to the manufacturer’s protocols (Santa Cruz Biotechnology, USA, Cell Signaling Technology, USA). After being washed three times with 1XPBS or 1XTBS (depending on the primary antibody) at room temperature, the blots were treated with the enhanced chemiluminescence reagent (Amersham; USA) to detect immuno-reactive proteins. The immunoblots were exposed to Biomax film (Koda;, USA) for visual representation. The band intensity was measured using ImageJ and graphed.

### 4.7. Immunochemistry

C2C12 cells were washed with phosphate buffered saline (PBS) (pH7.4) and fixed with 90% methanol at −20 °C for 10 min. After fixation, the cells were incubated in 5% milk in PBS for 30 min at 37 °C for blocking. Cells were incubated at room temperature with MF-20 (primary antibody) diluted in blocking buffer (5% milk PBS) for 1 h. After incubation, the cells were washed three times with PBS and incubated for 60 min at room temperature with a horseradish peroxidase (HRP)-conjugated α-mouse secondary antibody. The cells were washed again three times with PBS and incubated in developer (0.6 mg/mL DAB, 0.1 % H_2_O_2_ in PBS) to detect MyHC by immunocytochemistry. The nuclei were counter-stained with hematoxylin. Images were recorded with a microscope (Axiovert 35; Carl Zeiss MicroImaging) with either 4X NA 0.10 or 10X NA 0.25 Achrostigmat objective lenses with a digital camera (Canon, EOS D60; Japan).

### 4.8. Transcription Reporter Gene Assays

C2C12 myoblasts were transfected by a standard calcium phosphate-DNA precipitation method with the indicated reporter gene and expression constructs and pCMV-β-galactosidase to monitor transfection efficiency. After transfection, the cells were washed with PBS and maintained in GM and then treated as indicated. Total cellular protein was extracted with luciferase lysis buffer (20mM Tris-HCl pH7.4, 0.1% Triton X-100). Luciferase and β-galactosidase enzyme assays were performed according to the manufacturer’s protocol (Promega). Luciferase activity was quantified using a luminometer (Berthold Luma;, USA, 9501) and standardized according to β-galactosidase activity. Relative Luciferase units normalized for β-galactosidase activity (relative luciferase unit; RLU) were determined and plotted as an average of triplicate determinations and error bars represent standard deviations of the triplicate values.

## Abbreviations

TGFβ	Transforming growth factor beta
Smad	Mothers against decapentaplegic
MEK	Dual specificity mitogen-activated protein kinase kinase
ERK	Extracellular signal-regulated kinase
MyoD	Myoblast determination protein 1
